# Histone H3 Mutations in Cancer

**DOI:** 10.1007/s40495-018-0141-6

**Published:** 2018-04-26

**Authors:** Yi Ching Esther Wan, Jiaxian Liu, Kui Ming Chan

**Affiliations:** 1 Department of Biomedical Sciences, City University of Hong Kong, Hong Kong, China; 2 Key Laboratory of Biochip Technology, Biotech and Health Centre, Shenzhen Research Institute of City University of Hong Kong, Shenzhen, China

**Keywords:** Histone mutations, Cancer, H3K27M, H3K36M

## Abstract

Histone modifications are one form of epigenetic information that relate closely to gene regulation. Aberrant histone methylation caused by alteration in chromatin-modifying enzymes has long been implicated in cancers. More recently, recurrent histone mutations have been identified in multiple cancers and have been shown to impede histone methylation. All three histone mutations (H3K27M, H3K36M, and H3G34V/R) identified result in amino acid substitution at/near a lysine residue that is a target of methylation. In the cases of H3K27M and H3K36M, found in pediatric DIPG (diffuse intrinsic pontine glioma) and chondroblastoma respectively, expression of the mutant histone leads to global reduction of histone methylation at the respective lysine residue. These mutant histones are termed “oncohistones” because their expression reprograms the epigenome and shapes an oncogenic transcriptome. Dissecting the mechanism of H3K27M-driven oncogenesis has led to the discovery of promising therapeutic targets in pediatric DIPG. The purpose of this review is to summarize the work done on identifying and dissecting the oncogenic properties of histone H3 mutations.

## Introduction

Histones form the core of the nucleosome and are dynamically modified by epigenetic writers. Histone modifications include, but are not limited to, methylation, acetylation, phosphorylation, and ubiquitination. Being one form of epigenetic information, histone posttranslational modifications play an important role in regulation of gene expression. Therefore, the study of aberrant histone modifications in cancers has gained momentum in biomedical research recently.

Cancers are diseases stemming from misregulation of gene expression, and much of the research effort had been focused on dissecting the genetic alterations occurring in cancer cells. During the 1980s, biologists realized that cancers are caused not only by genetic mutations. Since 2000, it is well known that the histone methylation and acetylation are perturbed in cancer. These perturbations are often accounted to mutations in chromatin-remodeling proteins. Until recently, with the advance in sequencing technology, recurrent mutations on histones have been identified in several cancers. These histone mutations are implicated in oncogenesis and are therefore called “oncohistones.”

Current reports on oncohistones include H3K27M in glioma, H3K36M in chondroblastoma, and H3G34 mutations found in both glioma and bone cancers. Interestingly, these mutations are found on both H3.1 and H3.3. The former is a canonical histone, which incorporation onto the chromatin is restricted to S phase of the cell cycle. Meanwhile, H3.3 is a variant histone whose incorporation onto the chromatin is cell cycle independent [1] and is mediated by distinct histone chaperone complexes HIRA and DAXX–ATRX [2]. Contrary to H3.1 which is found throughout the genome, H3.3 localizes to the promoters and gene bodies of actively transcribed genes [2]. Despite having slightly different amino acid sequences, key lysine residues such as K4, K9, K27, and K36 are conserved on both H3.1 and H3.3.

All oncohistones reported so far have mutation on/near these conserved residues that are target of histone modifications. Expression of oncohistones impedes the deposition of histone marks and therefore reprograms the transcriptome that leads to oncogenesis. Although the precise oncogenic mechanism for some oncohistones remains to be uncovered, the discovery and characterization of oncohistones mark a milestone in cancer epigenetics. The purpose of this review is to summarize the work done on identifying and dissecting the oncogenic properties of histone H3 mutations: (1) H3K27M, (2) H3K36M, and (3) H3G34 mutations.

## H3K27M in Diffuse Intrinsic Pontine Glioma

Diffuse intrinsic pontine glioma (DIPG) arises from glial cells and is the most common form of brainstem glioma in children [3]. DIPG is an aggressive tumor with dismal prognosis; children diagnosed with DIPG have a median survival rate of 1 year. Recurrent somatic mutations on histone H3 that result in a lysine to methionine substitution on position 27 (H3K27M) were identified in up to 30% of pediatric glioblastoma patients and 60% specifically in DIPG [4–6]. These mutations occur mostly on *HIST1H3B* and *H3F3A*, the genes encoding histones H3.1 and H3.3 respectively, and were also identified in *HIST1H3C* and *HIST2H3C* with lower frequency [7, 8].

Interestingly, H3.1K27M tumors and H3.3K27M tumors form distinct subgroups in DIPG and have different clinical manifestations. First, H3.1K27M tumors are restricted to the pons whereas H3.3K27M tumors are also found along the midline of the brain [7–9]. Second, the two tumor types have their own set of associating DNA mutations. Mutations in *ACVR1* is associated with H3.1K27M [10] while amplifications of *PDGFRA*, *MYC*, *CCND2*, and *TP53* mutations are associated with H3.3K27M [9, 11]. Third, transcriptome analysis revealed different expression profiles in H3.1K27M and H3.3K27M subgroups. H3.1K27M tumors display a mesenchymal phenotype with genes associated with angiogenesis and hypoxia being upregulated. As for H3.3K27M tumors, they display an oligodendroglial phenotype. Lastly, the two types of tumors have different clinical outcomes; metastatic relapses are more frequently found in H3.3K27M patients and they have a shorter median survival of 11 months, compared to 15 months in H3.1K27M patients. In addition, H3.3K27M tumors are more resistant to radiotherapy [7]. Despite having different manifestations, expression of both H3.1K27M and H3.3K27M leads to similar alterations in the epigenome.

It is well documented that both H3K27me3 and H3K27ac play important roles in gene regulation. Trimethylation at histone H3 lysine 27 (H3K27me3) is a repressive histone mark associated with HOX gene silencing, X-chromosome inactivation, and genome imprinting, all of which are mediated by polycomb group (PcG) proteins [12]. EZH2, a subunit of the polycomb repressive complex 2 (PRC2), is the histone methyltransferase (HMT) responsible for H3K27me3 deposition. On the contrary, acetylation on H3K27 is associated with active gene transcription and enhancer regions. H3K27ac and H3K27me3 are mutually exclusive and H3K27ac is known to antagonize PRC2 activity [13]. The expression of H3K27M affects both methylation and acetylation on lysine 27 of Histone H3.

In addition to H3K27M, mutations on glycine 34 of histone H3 were also identified in brain cancers. The mutations lead to a glycine to valine/arginine substitution (H3G34V/R) and mark another clinically distinct group of DIPG. H3K27M and H3G34V/R mutations are mutually exclusive and are found in different regions of the brain [5].

### Alteration in Epigenetic Landscape

#### Alteration of H3K27me3 Landscape

Although the H3K27M mutation occurs only on 1 out of 16 genes encoding histone H3, it alters the epigenome of H3K27M-DIPG cells. Mass spectrometry revealed that H3K27M represents only 3to 17% of total H3 in DIPG patient cells [14]; its expression however reprograms the H3K27me3 landscape. On a global scale, dramatic reduction of trimethylated H3K27 was observed in H3.3K27M tumors [14–17], with concomitant gain of H3K27ac reported in some studies [14, 18, 19]. Nevertheless, enrichment of H3K27me3 was observed in hundreds of gene loci [15, 20]. Bender et al. (2013) noted that regions that lost H3K27me3 associated with promoters while regions that gain the mark associated with intergenic regions.

Loss of H3K27me3 upon H3K27M expression is a cell type-independent phenomenon. Chan et al. (2013) showed that the expression of H3.1K27M/H3.3K27M in human astrocyte, mouse embryonic fibroblast, and 293T recapitulated the reduction of H3K27me3 seen in DIPG cells. Lewis et al. (2013) reported similar findings and have demonstrated that incubation of H3K27M peptide with H3-WT nucleosome reduced the PRC2-mediated methylation by six folds, suggesting that H3K27M impedes PRC2 activity *in trans*. H3K27M exerts its effect by interacting with the SET-domain of EZH2. SET-domain is responsible for the methyltransferase activity of EZH2; its active site is lined with several highly conserved tyrosine residues. Substituting one of the tyrosine residues (Y641N) in the SET-domain resulted in loss of sensitivity to H3K27M inhibition, suggesting that the interaction between the hydrophobic methionine and aromatic tyrosine is responsible for the inhibitory effect of H3K27M. In line with their hypothesis, H3K27I/Nle (I: isoleucine; Nle: norleucine derivative) were also found to inhibit PRC2 activity.

The hypothesis was later confirmed by a structural study based on the crystal structure of K27M peptide-bound EZH2 [21]. Due to similarities in charge properties and configuration between the side chain of methionine and SET-bound lysine, methionine readily occupies the “lysine channel” lined with aromatic residues. Justin et al. (2016) reported 16-fold tighter binding between H3K27M peptide and PRC2 when compared to wild-type H3 peptide.

#### Gain of H3K27ac

**Fig. 1:**
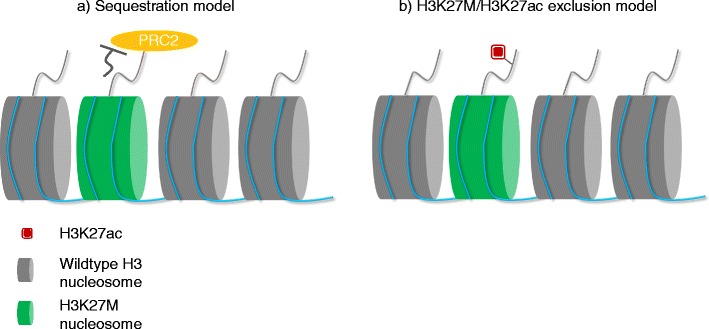
Models explaining the loss of H3K27me3 in H3K27M tumor. There are two models explaining the loss of H3K27me3 in H3K27M-expressing cells. In the sequestration model (**a**), it suggests functional inhibition of PRC2 complex by interaction with H3K27M nucleosome. ChIP-seq analysis by Piunti et al. later shows that H3K27M, instead of PRC2 subunits, co-localizes with H3K27ac. Their finding supports the H3K27M/H3K27ac exclusion model (**b**) where PRC2 is excluded from H3K27M/H3K27ac heterotypic nucleosome

Lewis et al. and Justin et al.’s work supports a sequestration model where H3K27M recruits and retains EZH2 locally and stops the spread of PRC2 complex, leading to global loss of H3K27me3 (Fig. 1a). A recent study however provides evidence against the sequestration model [19]. Piunti et al. (2017) studied the genome-wide distribution of H3K27M and found that SUZ12 and EZH2 (subunits of the PRC2 complex) are excluded from H3K27M-containing nucleosome. Instead, H3K27M localizes at actively transcribed region and coincides with H3K27ac and RNA polymerase II. Co-localization with H3K27ac is in line with a previous study in which H3K27M-containing oligonucleosome array was reported to have elevated level of H3K27ac [14]. Their work suggests that the loss of H3K27me3 is a result of PRC2 exclusion from the chromatin by H3K27M/H3K27ac nucleosome, instead of PRC2 recruitment by H3K27M (Fig. 1b).

### Alteration in Gene Expression

As an important player in long-term gene repression, alteration of H3K27me3 occupancy leads to concomitant alteration in gene expression. Gene ontology analysis on differentially expressed genes in H3K27M-expressing cells was shown to be involved in pathways in cancer, embryonic morphogenesis, and transcription factor activity [15], and neuronal differentiation [16]. Examples of differentially expressed genes are *PDGFRA* (platelet-derived growth factor receptor-α) and *MICA* (MHC class I polypeptide-related sequence A), which is upregulated and downregulated, respectively. *PDGFRA* had been implicated in gliomagenesis [22, 23], while downregulation of *MICA* is a potential mechanism for immune evasion in glioma. More recent study has implicated *CDKN2A* repression in H3K27M-driven tumorigenesis [20].

### H3K27M-Driven Oncogenesis

**Fig. 2:**
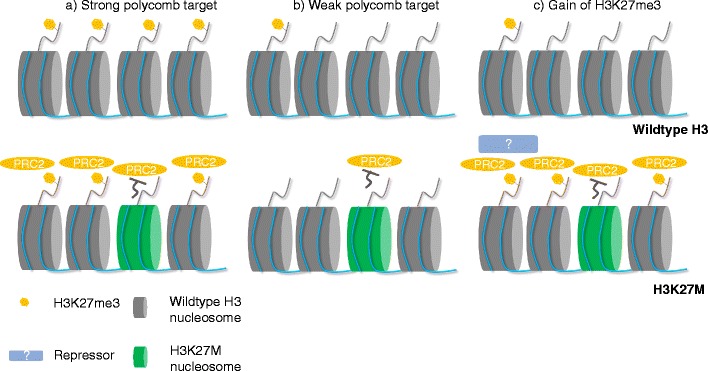
H3K27me3 landscape in H3K27M-expressing cells. H3K27me3 landscape at different loci. At strong polycomb targets enriched with H3K27me3 (**a**), the trimethylation mark remains even in the presence of H3K27M nucleosome. At weak polycomb targets where H3K27me3 is not abundant (**b**), incorporation of H3K27M leads to H3K27me3 loss. On the contrary, some loci gain H3K27me3 upon H3K27M expression (**c**). Mohammad et al. hypothesized that an unknown repressor recruits PRC2 complex to these loci

Despite global loss of H3K27me3 in H3K27M-expressing cells, the repressive histone mark is retained/enriched at some loci [15, 20]. Using a mouse model overexpressing *Pdgfβ*, Mohammad et al. (2017) reported that CpG islands (CGIs) enriched with H3K27me3 in wild-type H3 mouse tended to retain/gain H3K27me3 in the H3K27M counterpart. Since CGIs are known targets for polycomb complexes [24], it was suggested that loci that retained the mark are “strong” polycomb targets [20]. They therefore reasoned that the incorporation of H3K27M at strong polycomb targets is not sufficient to inhibit the overall PRC2 activity and thus the H3K27me3 level remains unchanged (Fig. 2a, b). Nevertheless, the mechanism by which some loci gain H3K27me3 remains elusive (Fig. 2c). It is however known that genes that gain H3K27me3 in H3K27M-DIPG are important players in maintaining the cellular identity of DIPG cells.

Gene ontology analysis of H3K27me3-enriched loci shows substantial enrichment of classic polycomb targets involving in regulation of transcription, pattern specification, and embryonic development. Interestingly, H3K27me3-assoicated genes in wild-type H3 neural stem cells overlap almost completely with that of H3K27M neural stem cells. In other words, there are few de novo polycomb targets in the PDGFB/H3K27M model, suggesting that H3K27M drives the oncogenesis of DIPG by reinforcing a transcriptional pattern specific for the cell-of-origin of DIPG [20]. However, a group of 20 genes that gain H3K27me3 in H3K27M mouse did not show H3K27me3 enrichment in H3 wild-type mouse, suggesting the silencing of these genes are selected for during the process of oncogenic transformation. Among this group of genes, *CDKN2A* is the most prominent gene that has been implicated in H3K27M-driven DIPG development [20].

*CDKN2A* encodes the tumor-suppressor p16 that halts the cell cycle during cellular stress and oncogene activation [25]. While homozygous deletion of *CDKN2A* is found in > 55% of adult high-grade glioma [26], it is rarely seen in pediatric high-grade glioma. Thus, PRC2-mediated gene repression could be an alternative pathway to silence *CDKN2A* expression [27]. By inducing expression of p16, DIPG cell lines showed growth arrest, demonstrating that the growth of DIPG is dependent on repression of *CDKN2A* [20]. Repression *CDKN2A* was later reported to accelerate gliomagenesis in a H3.3K27M mouse model [28].

### *p16*-Independent Oncogenesis

Nevertheless, it is important to note that *CDKN2A* silencing is not the only H3K27M-driven oncogenic mechanism. As mentioned, H3K27M localizes at actively transcribed regions, in addition to H3K27ac and RNA polymerase II, H3K27M also coincide with that of BRD2 and BRD4, bromodomain and extra-terminal domain (BET)-containing proteins. To dissect the role of BRD2 and BRD4 in H3K27M-DIPG cells, JQ1 was used to inhibit the two BET domain-containing proteins. In line with the role of BRD2/4 in transcription elongation, JQ1 treatment shuts down many actively transcribed genes. Intriguingly, genes affected by JQ1 are occupied by BRD2, BRD4, and H3K27M, indicating that these genes are direct targets. Most importantly, the treatment demonstrated anti-tumor effect without de-repression of *CDKN2A* [19], raising the possibility of a p16-independent oncogenic pathway.

Piunti and colleagues also reported H3K27M occupancy at active-enhancer region (marked by H3K4me1 and H3K27ac), suggesting that H3K27M might drive oncogenesis by participating in the formation of super-enhancers.

Last but not least, studies in mouse model also suggest that H3.3K27M-mediated oncogenesis also depends on other co-occurring mutations. In the absence of *Trp53*, *PDGFRA* overexpression and *ATRX* knockdown in H3.3K27M-expressing mouse decrease tumor latency and promote focal tumor formation, respectively [29]**.**

### Therapeutic Targeting of H3K27M-DIPG

Although the precise mechanism of H3K27M-driven oncogenesis is yet to be elucidated, research effort has led to the discovery of several druggable targets in H3K27M-DIPG. Approach taken by each study is vastly different from each other, but several small molecules have shown therapeutic efficacy against H3K27M-DIPG both in vitro and in vivo.

#### Rescuing H3K27me3

As reduction of H3K27me3 is the most prominent consequence of H3K27M expression, rescuing the level of H3K27me3 is therefore the most intuitive approach in developing targeted therapies for K27M-DIPGs. GSKJ4, a demethylase inhibitor, was reported to have anti-tumor activity both in vitro and in vivo [30]. In mouse xenograft model, GSKJ4 retarded the growth of tumor. GSKJ4-treated human H3K27M-DIPGs cell lines showed lower viability, reduced colongenic activity, and are more apoptotic. Hashizume et al. (2014) further demonstrated that the anti-tumor effect of GSKJ4 is due to inhibition of JMJD3, the demethylase for H3K27me3 [31]. The working mechanism is however unclear as GSKJ4 has also shown to inhibit demethylases of other families [32]. A more detailed analysis on the transcriptome of GSKJ4-treated cells is required to dissect the effect of GSKJ4.

#### Reversing the Gain of H3K27ac

As reported by different studies, the loss of trimethylation at K27 leads to concomitant gain of acetylation at K27 [7, 8], leading to the idea of reversing this epigenetic alternation as an alternative potential therapeutic approach. Panobinostat is a HDAC inhibitor that has shown therapeutic efficacy in H3K27M-DIPGs [33]. Panobinostat-treated K27M-DIPG cell lines have lower viability and are more apoptotic. Interestingly, in addition to rescuing H3K27me3 level, there was an unexpected increase of H3K27ac. Gain of H3K27ac could be explained by the finding that acetylation occurring on the same H3 tail (where H3K27M is situated) could relieve the inhibition of K27M towards EZH2 [34]. Grasso and colleagues further demonstrated the downregulation of *MYC* target genes upon panobinostat treatment. It is also known that panobinostat and GSKJ4 can act synergistically to reduce the viability of H3K27M-DIPG cells [33].

JQ1, an inhibitor of BET, showed anti-tumor activity in both DIPG cell lines and xenograft model [19]. While its working mechanism remains unknown, JQ1 lowers H3K27ac level in a time-dependent manner. Moreover, JQ1 upregulates *p21*(cell cycle arrest marker), *TUBB3*, and *MAP2* (marker for differentiation in mature neurons), resulting in its anti-proliferative effect and neuron-like morphological changes in H3K27M-DIPG cells.

#### Targeting Residual EZH2 Activity

Inhibition of EZH2 sounds like a counter-intuitive approach in treating DIPG, but studies have demonstrated that the residual EZH2 activity is important for the growth of DIPG cells [19, 20]. H3K27M-DIPG cell lines treated with EPZ6438, a highly selective EZH2 inhibitor, showed reduced proliferation and colony-forming ability. It is believed that the anti-proliferative effect is mediated by de-repression of *p16*, as demonstrated by the loss of drug sensitivity upon *p16* [20] knockdown. It is however possible that EPZ6438 could act through a p16-independent pathway. Although both SF8628 (H3.3K27M) and DIPG-IV (H3.1K27M) showed reduced growth upon *SUZ12*/*EED* knockdown, p16 was only upregulated in SF8628 [19].

DIPG cells treated with JQ1 and EPZ6438 show upregulation of *p21* and *p16* respectively, two important cell cycle regulators. It would be interesting to investigate whether the use of JQ1 and EPZ6438 together could suppress the growth of DIPG cell lines synergistically in vitro and in vivo.

## H3K36M in Chondroblastoma

Chondroblastoma is a cartilaginous tumor found in children and adolescents [35]. Soon after the report of H3K27M in pediatric DIPG, H3.3K36M mutation was discovered in 73/75 (95%) of chondroblastoma samples. Interestingly, 68 cases of K36M were found on *H3F3B*, one of the two genes that code for the variant histone H3.3. The mutation is also tumor type-specific as screening of seven additional bone cancers reveals low prevalence of H3.3K36M [36]. H3K36M mutation has also been reported in head and neck squamous cell carcinoma [37] and colorectal cancer [38].

### Perturbation of H3 Methylation and HMT Inhibition

Global reduction of H3K36 methylation was observed in chondroblastomas harboring the H3.3K36M mutation. ChIP-seq analyses of H3.3K36M cells demonstrated H3K36me2 depletion in intergenic regions [39, 40], whereas the loss of H3K36me3 occurred throughout gene bodies and was correlated with loss of H3K36me2 [39]. Unsurprisingly, this global loss of K36 methylation is a result of histone methyltransferase inhibition, as supported by enrichment of HMTs in pull-down using H3K36M-containing mono-nucleosome [39, 40].

The inhibitory effect of H3K36M towards HMT was supported by solving the crystal structure of SETD2-bound K36M peptide [41, 42]. The binding of K36M peptide to SETD2 is similar to that of K27M to EZH2, where the inhibitory effect is also mediated by interaction between tyrosine residues in the SET-domain and the mutated methionine.

Knockdown experiment showed that inhibition of NSD2 and SETD2 is responsible for the loss of H3K36me2/3 respectively. In addition to the loss of H3K36me3, Lu et al. (2016) reported the gain of H3K27me3 upon Nsd1/Nsd2/Setd2 triple knockdown in murine mesenchymal progenitor cells (MPC). Gain of H3K27me3 was also reported in 293T and murine MPC expressing H3K36M. Since H3K36me2/3-containing nucleosomes are poor PRC2 substrates in vitro [43, 44], the loss of H3K36 methylation provided new substrates for PRC2, resulting in increase of H3K27me3.

### De-repression of PRC1 Target Genes

ChIP-seq of H3K36M-MPC revealed the enrichment of H3K27me3 in intergenic regions, but an overall decrease in gene-associated-to-intergenic-H3K27me3 ratio [40]. This suggests a re-distribution of the H3K27me3 and possibly PRC1, the reader for H3K27me3. Indeed, recruitment of PRC1 was altered in H3K36M MPC, with a dramatic decrease of Ring1b and Cbx2 (subunits of PRC1 complex) binding at H3K27me3-bound genes. In addition, mono-ubiquitination on H2AK119 (catalyzed by PRC1) was found to be enriched in intergenic regions that gained H3K27me3, indicating PRC1 spreading in those regions. This “dilution” of PRC1 away from repressed gene loci led to upregulation of PRC1 target genes in H3K36M cells. Examples of de-repressed genes include *Wnt6* and *Sox6* which are associated with self-renewal and lineage specification of mesenchymal stem cells. De-repression of PRC1 target genes are in line with the differentiation blockade observed in H3K36M-expressing murine MPC.

It is however plausible that H3K36M drives oncogenesis by some other non-mutually exclusive mechanisms. In addition to differentiation blockade, other oncogenic properties have been reported in H3K36M cells. Fang et al. (2016) demonstrated that human chondrocytes expressing H3K36M are more colongenic and are more resistant to staurosporine-induced apoptosis. They have also demonstrated downregulation of genes involved in chondrocytic differentiation (*BMP2* and *RUNX2*) and homologous repair (*BRCA1* and *ATR*).

### Therapeutic Targeting of H3K36M Chondroblastoma

A WEE1 kinase inhibitor (AZD1775) has been shown to selectively kill H3K36me3-deficient cancer cells [45]. In H3K36me3-deficent U2OS cells, sensitivity towards WEE1 inhibitor is mediated by *RRM2*. *RRM2* encodes a subunit of ribonucleotide reductase; the degradation of its protein product is regulated by CDK/SCF^cyclin F^, which is negatively regulated by WEE1. It was discovered that *RRM2* is downregulated in H3K36me3-deficient cells, leading to depletion of the cellular dNTP pool. Under the concept of synthetic lethality, WEE1 inhibitor treatment would lead to unregulated degradation of RRM2 by CDK/SCF^cyclin F^, further depleting the cellular dNTP pool. This approach might be used to treat H3K36M chondroblastoma, where H3K36me3 is lost on a global scale. The efficacy of WEE1 inhibitor against K36M tumor however needs further validation.

## H3.3G34 Mutations

Mutations on H3.3G34 were found in glioblastoma (G34R/V) [4, 6], giant cell tumor of the bone (G34W/L), and osteosarcoma (G34R/V) [36]. Brain tumors harboring H3K27M and H3G34V/R are found in different anatomical locations. While most H3K27M tumor are found along the midline (pons, thalamus, and the spinal cord), H3.3G34V/R tumors usually arise in the cerebral cortex [5]. Moreover, Sturm and colleagues have also demonstrated that the two mutations are age group specific. H3K27M are found predominantly in children (median age, 10.5), and G34R/V are found in adolescent (median age, 18).

In contrast to H3K27M and H3K36M that act *in trans* to cause global reduction of methylation at their respective residue, H3G34 acts *in cis*. Overall reduction of H3K36me2/3 was not observed when epitope-tagged H3.3G34V/R histones were expressed in 293T cells. Instead, it caused reduction of H3K36me3 only on nucleosome containing the G34➔V/R histone H3 [14] and expression of H3.3G34V/R in yeast yields the same result [41]. Interaction analysis indicates that G34 is involved in the recognition of H3 by SETD2 [42]. SETD2 has a small pocket for G34 on histone H3, so small that only glycine could fit in. As a result, any substitution on G34 would abolish the recognition of H3 by SETD2, thus reducing H3K36me3 on that particular histone.

### H3.3G34V/R in Pediatric High-Grade Glioma

Compared to H3K27M and H3K36M, little is known about the role of H3.3G34 mutations in oncogenesis. It is known that H3.3G34V expression leads to differential distribution H3K36me3 on around 150 genes. Gene ontology analysis of these differentially bound genes showed enrichment in forebrain development, neural differentiation, and cell proliferation. Interestingly, *MYCN* is the most differentially bound and is amplified in G34V-GBM cells [46]. Its role in shaping the transcriptome of G34V/R-GBM cells has yet to be elucidated.

### H3.3G34W/L in Giant Cell Tumor of Bone

G34W and G34L occur in 92 and 2% of giant cell tumor of bone patients, respectively [36]. Primary cell lines expressing H3.3G34W has higher proliferation, migration, and colony formation. Transcriptome analysis also revealed aberrations in RNA processing, such as exon skipping and exon inclusion [47].

### Therapeutic Targeting of H3.3G34V/R in DIPG

H3.3G34V/R is frequently associated with *AKT1* amplification [9]. Dual inhibition of PI3K/AKT and MEK/ERK pathways has been shown to reduce viability of DIPG cell lines in a synergistic manner [48].

## Perspective

Since the first report on recurrent H3K27M mutation in glioma [4–6], the roles of histone mutations in tumorigenesis have started to surface. So far, the best characterized oncohistone is H3.3K27M in DIPG. Traditionally, pediatric glioma and adult glioma were treated with the same regimen. However, the discovery of pediatric glioma-exclusive H3K27M supports the notion that pediatric gliomas and adult gliomas are fundamentally different on a molecular level and require different treatment. Understanding the mechanism of H3.3K27M-driven oncogenesis has led to the discovery of potential therapeutic targets in DIPG.

Although great progress has been made, many questions remain unanswered. Of the three oncohistones reported, only H3K27M is found on both H3.1 and H3.3. The former is ubiquitously expressed and is distributed uniformly in the genome. In reality, H3.1K27M has only been identified in DIPG; it is therefore unclear what contributed to the tumor-type specificity of H3.1K27M. In general, the tumor type-specific occurrence of oncohistones remains largely unknown.

As mentioned in the Introduction, it was long known that histone methylations are perturbed in cancers and alterations in histone methyltransferases have been identified in multiple cancers. For example, inactivating mutations of EZH2 have been found in follicular lymphoma and diffuse large B cell lymphoma [49]. However, H3K27M occurs only at low frequency in follicular lymphoma (1/415) [50]. Similarly, H3K36M has not been reported in renal cell cancer where SETD2-inactivating mutations were identified [51]. Since inactivation of both EZH2 and SETD2 leads to aberrant methylation at the respective histone H3 lysine residue, it is unclear why H3K27M and H3K36M are not as frequent as expected in these cancers. One possibility is that oncohistones confer addition function(s) that alter gene regulation in addition to specifically inhibiting the corresponding histone-modifying enzyme(s), and/or inhibit other enzymes.

Last but not least, it is important to note that histone modifications are not restricted to lysine methylation on histone H3. Examples of other histone posttranslational modifications include serine/threonine phosphorylation, lysine acetylation, and ubiquitination. Mutations in the writers, readers, and erasers of these histone tail modifications were identified and have been implicated in cancers [52]. Could this point to the existence of the corresponding histone mutations? Moreover, as histone core modifications started to gain attention, it is likely that more oncohistones will be identified in the future.
